# Dual-Scale StaphAIR: Predictive Modeling for the Diagnosis of *S. aureus* Infection via Simultaneous Detection and Quantification of Cytokines and Antibodies

**DOI:** 10.1021/acs.analchem.6c00764

**Published:** 2026-05-16

**Authors:** Alanna M. Klose, Christopher A. Beck, Stephen L. Kates, Gowrishankar Muthukrishnan, Edward M. Schwarz, Benjamin L. Miller

**Affiliations:** † Department of Dermatology, 6927 University of Rochester, Rochester, New York 14627, United States; ‡ Center for Musculoskeletal Research, University of Rochester Medical Center, Rochester, New York 14627, United States; § Department of Biostatistics and Computational Biology, University of Rochester Medical Center, Rochester, New York 14627, United States; ∥ Department of Orthopaedic Surgery, 6886 Virginia Commonwealth University, Richmond, Virginia 23284, United States

## Abstract

*Staphylococcus aureus* causes life-threatening bacterial infections. Current diagnostics for bone infection consist of invasive sample collection and lengthy microbial culture; available blood tests are not effective in the orthopedic context. To address this gap, we report an immune biomarker panel for the simultaneous measurement of cytokines and *S. aureus*-specific antibodies in serum and evaluate predictive machine learning models for the diagnosis of *S. aureus* infection. The approach relies on a newly developed “dual scale” 25-plex Arrayed Imaging Reflectometry (AIR) immunoassay able to simultaneously quantify 6 cytokines and 19 *S. aureus*-specific antibodies on a single platform. The dual-scale StaphAIR assay was used to quantify 429 individual serum samples collected from patients hospitalized with culture-confirmed *S. aureus* infection (infected) and from healthy patients undergoing elective surgery (control). Multivariate logistic regression was used to identify combinations of predictors with the highest diagnostic potential. We found that the most important single predictor was IL-6 (AUC 0.85), and the highest-performing logistic regression combination was IL-6 + IL-10 + IL-17A + TNFα + IsdB (AUC 0.92). Patient demographic data (age, body mass index, race, gender, smoking status) and laboratory data (hemoglobin A1c and albumin) were assessed, and only albumin was found to be a potential confounder. Then, predictive machine learning models were trained and validated (5-fold cross-validation) using the dual-scale StaphAIR data with and without albumin as a predictor and evaluated using Receiver Operating Characteristic and Area Under the Curve (ROC/AUC) analysis. The neural boosted model performed the best on the validation folds with and without albumin level as a variable (AUC 0.975 with albumin, 0.914 without). Predictive models using combinations of immune biomarkers in serum demonstrated excellent diagnostic utility for *S. aureus* infection, although further external validation with an independent data set is recommended. We anticipate that this approach will also have significant value in diagnosis of other bacterial or viral infections.

## Graphical abstract

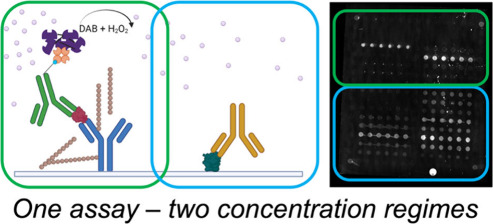

The infection rate due to methicillin-resistant *S. aureus* (MRSA) and methicillin-sensitive (MSSA) strains in the United States has proved difficult to decrease below about 50 cases per 100,000 individuals.^1^ Surgeons have improved aseptic techniques,^2^ screening procedures,^3,4^ and preop protocols^5^ to decrease infection rates, but this has not eliminated the problem.^6^ Many infections are highly virulent, difficult to treat, and can become recurrent. Diagnosis of *S. aureus* infection currently requires a culture-positive sample, which is time-consuming and invasive.^7^ A blood-based test that measures immune biomarkers for diagnosis of *S. aureus* infection could be a rapid, noninvasive tool to guide treatment and enable longitudinal monitoring.

*S. aureus* is a formidable pathogen because it has an arsenal of virulence factors enabling evasion of the human immune system by blocking complement, interfering with neutrophil function, blocking opsonization, stimulating the production of nonprotective antibodies, aiding endosomal escape, and dysregulating T-cell pathways, among other functions^8^ (Table 1). The detection of antibodies against *S. aureus* virulence factors introduces pathogen-specificity, and has shown good ability to discriminate between control and infected groups using Luminex^7^ and Arrayed Imaging Reflectometry (AIR) assays,^9^ with a multivariate logistic regression model using data from an 8-plex “StaphAIR” array achieving an AUC > 0.85. The cytokine IL-6 has been described as an effective biomarker for diagnosing infection, but it does not provide information about the causative pathogen.^10^ Both the innate and adaptive arms of the immune response are essential in the battle against *S. aureus* in both acute and chronic infection.^11,12^ Both cytokine and antibody levels have been evaluated in studies of the immune response to other pathogens including RSV, SARS-CoV-2, and Influenza.^13–15^ Furthermore, work measuring an immune signature comprised of cytokine response and *S. aureus*-specific antibody levels found utility in both biomarker types for differentiating between patients with infections and healthy controls. However, that work required 4 different immunoassay techniques including a custom *S. aureus* protein screening microchip, Luminex, ELISA, and a Meso Scale Discovery cytokine panel to measure cytokine and antibody biomarkers.^16^ Detecting both cytokine and antibody response is clearly important when studying the immune response to infectious pathogens. To this end, the ability to use a single technique could improve assay consistency, decrease sample volume requirements, and improve clinical accessibility, in addition to reducing costs. The objective of this study was to demonstrate that a newly developed dual-scale StaphAIR, capable of simultaneous cytokine and antibody detection over a broad analytical range, in combination with predictive models for the diagnosis of *S. aureus* infection, could meet that need.

**Table 1:** *Staphylococcus aureus* Virulence Factors on the Dual-Scale StaphAIR Array

Immune evasion mechanism	Virulence factor name	Virulence factor action	Abbreviation
Biofilm Formation	Iron surface determinant proteins A, B, and H	scavenge iron from heme	IsdA, IsdB, IsdH
	Autolysins (amidase, glucosaminidase)	enzymes for cell-wall splitting	Amd, Gmd
Superantigen T-cell activation	Staphlyococcus enterotoxin A, B, C and enterotoxin-like I, Q,X, Toxic shock syndrome toxin-1	bypass antigen presentation and tether MHC class II receptor and T-cell receptor	SEA, SEB, SEC, SelI, SelQ, SelX, TSST-1
Neutrophil dysregulation	Staphyloccous enterotoxin-like X	interferes with neutrophil extravasation	SElX
	Panton-Valentine Leukocidin subunits F and S	pore forming toxins lyse neutrophils	LukS, LukF
	Chemotaxis Inhibitory Protein of *Staphylococcus aureus*	interfere with chemotaxis	CHIPS
	Alpha-toxin or alpha hemolysin	lyses neutrophils by forming pores in cell membrane	α-toxin/Hla
	Staphylococcal Complement Inhibitor	intereferes with all three compement pathways	SCIN
Reduced T-cell receptor diversity	alpha-toxin or hemolysin alpha	impairs dendritic cells and reduces antigen presentation to T-cells	α-toxin/Hla

AIR is a label free biosensing technique that measures increased thin-film thickness due to analyte binding to spatially multiplexed ligands on the Si/SiO_2_ chip surface.^17^ The technical advance of dual-scale StaphAIR is the simultaneous detection of cytokines and antibodies achieved by mass-based amplification of the low abundance (pg/mL) cytokine response alongside unamplified (label-free) high abundance (μg/mL) antibody response, a 1 million-fold difference of concentration, in a single sample dilution. This development also addresses a broader analytical need for simultaneous measurement of analytes across a very wide dynamic range of concentrations.

## Experimental Section

### Serum Samples

This retrospective study used 429 serum samples collected before December 2019; details of sample acquisition and handling were approved by the ethics review board of the Virginia Commonwealth University and were previously reported by a separate study.^18^ The sample aliquots were shipped to the University of Rochester under a materials transfer agreement. 246 samples were from hospitalized individuals with culture-confirmed *S. aureus* infection, and 183 samples were from uninfected control patients scheduled for elective surgery. Other information included culture results, resistance or sensitivity to methicillin, infection location (bone, pulmonary, endocarditis, bacteremia of unknown origin), patient demographic data (age, body mass index, race, gender, smoking status), laboratory data (hemoglobin A1c and albumin), and information about one-year outcomes (adverse, infection controlled, unknown). Adverse outcomes included death, persistent infection, amputation, and joint fusion. We note that although these samples are not sufficient to adequately test differential diagnosis of *S. aureus* infection given that the set did not include samples from patients testing culture-positive for other bacterial infections, we anticipated that they would be adequate for the purposes of this study. Array developmental work was performed using a complex serum matrix composed of serum samples collected under a University of Rochester Institutional Review Board-approved protocol from healthy volunteers.

### *S. aureus* Antigens and Cytokine Capture and Detection Antibodies

Recombinant *S. aureus* antigens IsdA, IsdB, and IsdH, Gmd, Amd, CHIPS, and SCIN were selected and produced by Genscript as described previously,^9^ except without the biotinylated AviTag. These full-length proteins represent different classes of *S. aureus* virulence functions (Table 1). This study employed virulence factors with diverse functions because this has been shown to improve the discriminatory potential of logistic regression models for infection.^19^ The LukS and LukF were tag free and purchased from IBT Bioservices (LukS #:0540-001, LukF#:0540-002). SEA, SEB, SEC, SelI, SelX, and SelQ and toxins TSST-1 and alphatoxin/HemolysinA (HLA) were gifts from Dr. Patrick Schlievert and were stored and handled in accordance with the approval and guidelines of the University of Rochester Biosafety Office. The capture and detection antibodies for CRP, procalcitonin (PCT), IL-6, IL-10, IL-17A, IL-17F, IL-27, and TNFα were purchased from commercial suppliers (Table S1). The goat polyclonal antibody against Fluorescein (FITC, Rockland #600-101-096) was not fluorescent and was used as a negative control because there should not be any fluorescein in human serum.

### AIR Substrate Preparation

Polished silicon wafers with ∼1400 Å of thermally grown oxide were diced into 5 × 5.75 mm chips. Batches of ∼200 chips with ∼1424 ± 1 Å oxide thickness underwent a NaOH/EtOH wash (7:3 solution of ethanol and 10 M NaOH for 30 min) to remove dicing debris; they were then etched in hydrofluoric acid to ∼1412 Å, rinsed with water, and dried with N_2_ gas before being chemically functionalized with ∼5–10 Å of amine-reactive (3-glycidyloxypropyl) trimethoxysilane (GPTMS (Sigma-Aldrich, 440167)) in a plasma process chemical vapor deposition system (YES 1224P) at the University of Rochester Integrated Nanosystems Center.

### Array Fabrication

IsdA, IsdB, IsdH, Gmd, SCIN, Amd, and CHIPS, capture antibodies αIL-10, αIL-17A, αIL-27, and the αFITC correction probe were dialyzed using 50 μL volume in 30 kDa molecular weight cutoff slide-a-lyzer dialysis cups floating in a beaker of PBS (137 mM NaCl, 2.7 mM KCl, 10 mM Na_2_HPO_4_, 1.76 mM KH_2_PO_4_–H_2_O, pH 7.4) stirred slowly with a magnetic stir bar for 1.5 h, RT. This was done to remove components from the stock solutions including glycerol, Tris-HCl, and sodium azide that interfere with protein attachment to the chip surface.^20^ All probe solutions contained 2% trehalose as an additive to improve spot morphology.^21^ Optimized print solutions used to produce all arrays are shown in Table S2. A sciFLEXARRAYER SX (Scienion A.G.; Berlin, Germany) piezoelectric printer with a PDC70 capillary with type 4 coating was used to print an array of probe spots (Figure S1) onto chips mounted onto aluminum jigs inside a controlled humidity chamber at 85% ± 4% relative humidity. Droplets were between 300 and 350 pL in volume, as measured by the instrument, and a single droplet was used for each probe spot. Each probe was arrayed with 6 replicate probe spots. The chips remained in the humidified chamber overnight to ensure covalent attachment of the protein antigens to the GPTMS layer.

### Blocking and Stabilizing AIR Arrays

Each jig containing ∼100 arrayed chips was removed from the humidified microspotting chamber and immediately placed sideways into a custom 3D printed well containing 300 mL of 50 mM NaOAc, pH 5.0, for 5 min to prevent smearing of any noncovalently bound probes onto nearby areas of amine-reactive chip surface (Figure S2). The jigs were then incubated in 1% Bovine Serum Albumin (BSA; Rockland #BSA-50) w/v in NaOAc, pH 5.0, for 30 min to block the background from nonspecific adsorption, and then incubated in a second blocking solution of 20% fetal bovine serum (FBS; Gibco, #A5670701) in Assay Wash Buffer (AWB: mPBS with 0.005% tween-20, pH 7.2) for 30 min. Following blocking, the jigs were submerged in AWB and incubated for 5 min before being transferred to StabilCoat Plus (Surmodics, Inc.) for 30 min. All incubation steps were performed at RT with 80 rpm orbital shaking. Only one jig could be processed at a time, and it was transferred between two wells to prevent the arrays from drying out at any point during the process. The blocked arrays were placed into a 40 °C oven for 30 min before being loaded into low-volume plastic consumables.

### Loading Arrayed Chips into Low-Volume Consumables

Custom adhesive sheets were applied to an entire 96-well plate worth of consumable bases at a time (Figure S2). Each base was mounted into a plastic strip that snaps into a plastic 96-well plate. The back of each AIR array was cleaned with a Nanopure-soaked Kimwipe and dried with a cleanroom wipe before being attached to the adhesive. A good seal is essential to prevent the sample from leaking out of the consumable underneath the chip. The adhesive had three holes open so that the chip can lay flat on the corresponding 3 pins in the Adarza ZIVA AIR instrument (Adarza BioSystems, Inc.) to ensure that it is flat at the correct imaging plane. A hole was punched into the consumable cap using scissors prior to the cap being snapped onto the consumable base. The addition of a larger hole helped to decrease the size and prevalence of bubbles on top of the AIR chip lying flat in the consumable. Completed arrays in consumables were stored in a sealed mylar bag with desiccant and nitrogen gas at 4 °C until use in immunoassay experiments. All assays with serum were completed within one month of array production.

### Dual-Scale StaphAIR Array Amplification of Low-Abundance Analytes

The detection of antibodies bound to *S. aureus* virulence factors on the array was unamplified and label-free, while a series of postincubation steps were conducted to amplify the reflectance signal for low-abundance analytes. A simplified diagram showing the stepwise mass-based amplification process is shown in Figure 1. Incubation with sample occurred overnight at 4 °C, and the amplification steps were performed at room temperature the next morning. The sample was aspirated out of the low-volume consumable and replaced with EAB wash buffer two times (Enhanced Assay Buffer, EAB: 20 mM Tris Base, 250 mM NaCl, 250 mM KCl, 3% w/v propylene glycol, 0.125% Triton X-100, and 1% w/v BSA). Then, commercial biotin-conjugated detection antibodies (Table S1) (1 μg/mL each of IL-6, IL-17A, IL-10, IL-27, and PCT) and 2 μL/mL each of αTNF-α and αIL-17F were mixed together and incubated with the arrays for 30 min, shaking at 420 rpm on a plate shaker. The caps were then removed from the consumables, and the arrays were flipped upside down into 3D printed wells full of EAB wash buffer on a shaker at 200 rpm for 3 min (Figure S3). The wells were designed to ensure that each array was fully submerged without touching the bottom of the well. Then, the arrays were moved to wells containing 1 μg/mL streptavidin poly horse radish peroxidase (SA-poly-HRP) protein conjugate (ThermoFisher #N200) in EAB, 200 rpm, for 15 min followed by 3 min in EAB before being incubated for 10 min, shaking at 200 rpm, in a mixture of 3,3′-diaminobenzidine (DAB) substrate in hydrogen peroxide prepared according to kit instructions (DAB kit, ThermoFisher #34002). HRP catalyzes the polymerization of DAB, which deposits locally to increase the thickness of the probe spot. The arrays were transferred to wells containing Nanopure water to remove residual DAB mixture before they were washed under a stream of Nanopure water and dried under a stream of nitrogen gas. All serum aspirate fluid was treated with bleach according to BSL2 protocol. Any tween-20 or triton-X-100-containing fluid (AWB and EAB) and DAB waste was disposed of as hazardous waste according to standard University of Rochester Environmental Health and Safety protocols.

**Figure 1:**
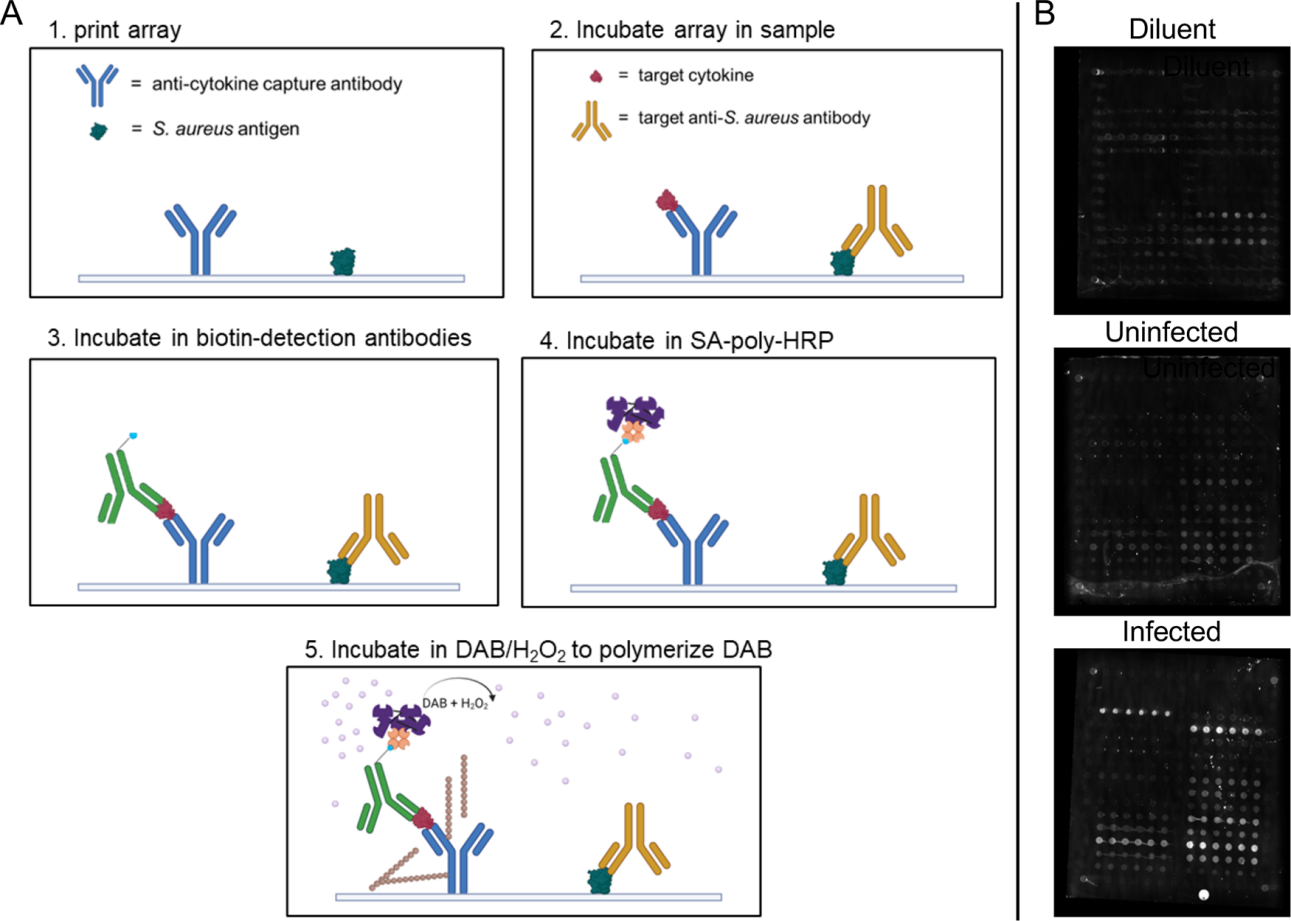
Simplified diagram of the mass-based amplification process for target cytokines. (A) The process is a sandwich assay using biotin-conjugated detection antibodies which bind streptavidin poly-HRP. Mass amplification occurs due to localized deposition of polymerized DAB substrate catalyzed by HRP in the presence of hydrogen peroxide. (B) AIR images of arrays incubated in diluent, uninfected serum, or infected serum. All arrays underwent the full amplification protocol.

### Dilution Curves, LOB, LLOD, LLOQ, and ULOQ Calculations

Two sets of cytokine dilution curves were evaluated. One was in a simple EAB matrix. The other was in a more complex serum matrix comprised of a 1:5 dilution of a pooled serum sample from healthy volunteers, collected under a University of Rochester Institutional Review Board approved protocol, in EAB with 30% FBS (EAB30). The FBS was included to block observed assay interference likely due to heterophilic antibodies in the serum samples (Figure S4). Commercially available recombinant proteins were mixed and serially diluted in matrix. Each dilution, including an EAB-only “Blank”, was incubated with a separate dual-scale StaphAIR array in triplicate overnight and received the full amplification protocol the next morning. The EAB matrix curves were prepared using the same target cocktail mixture, batch of StaphAIR chips, and analyzed on the same day. The complex serum matrix was prepared with a 1:5 dilution of pooled human serum from uninfected individuals in EAB with 30% FBS, and each replicate curve was evaluated on a different day, with a different chip production batch and different preparation of target cocktail. Average thickness values were plotted with Matplotlib, and numerical analysis was performed with NumPy, Pandas, and SciPy (Python 3.12.11) using Spyder 6.1.2. The error bars are the standard deviation of thickness values measured on triplicate chips. A 4-parameter logistic (4PL) regression fit was applied and used to convert thickness values to cytokine concentration in pg/mL based on results from calibration experiments with recombinant proteins. In this regression (eq 1), *A*_1_ is the minimum thickness on the curve, *A*_2_ is the maximum thickness on the curve, *x*_0_ is the inflection point, and *p* is the slope of the curve at the inflection point.

1 $$y=A_{2}+\frac{A_{1}-A_{2}}{1+{\left(\frac{x}{x_{0}}\right)}^{p}}$$

The limit of the blank (LOB) was calculated from the control values for each antigen (eq 2), where μ is the mean, and σ is the standard deviation. The lower limit of detection (LLOD) was calculated from a combination of all low-concentration thickness values and standard deviations (near and below the inflection point of the logistic curve) (eq 3).^22–24^

2 $$\mathrm{LOB}=\mu_{\mathrm{blank}}+1.645\left(\sigma_{\mathrm{blank}}\right)$$

3 $$\mathrm{LLOD}=\mathrm{LOB}+1.645\left(\sigma_{\mathrm{low‐concentration samples}}\right)$$

The LOB was calculated from the arrays incubated in matrix only. The lower limit of quantitation (LLOQ) was defined as the lowest real concentration of recombinant protein measured with a coefficient of variation (CV) of less than 20%. The upper limit of quantitation (ULOQ) was defined as the highest concentration with CV < 20% and a mean signal not within 5% of the upper asymptote of the fitted 4PL curve. The analytical range was defined as all concentrations between the LLOQ and ULOQ. The LOB, LLOD, ULOQ, and ULOQ for simple EAB matrix 4PL fit (Figure S5) and complex serum matrix 4PL fit (Figure S6) are presented in Table 2. The LOB, LLOD, and LLOQ values are higher in complex serum matrix compared to simple EAB matrix as expected due to interference from protein background, immunoglobulin content, complement, and inflammatory proteins in real human serum samples. The standard deviation was higher between replicate curves in the serum matrix, potentially due to variables including target cocktail mixture and other handling differences. Antibody dilution curves constructed from serially diluted pooled serum from individuals with a culture-confirmed *S. aureus* infection are shown in Figure S7. As expected, the antibody response in these samples decreases with dilution factor. Some probes not shown (SEA, SEQ, SelI, SEC) did not have the expected response on this early version of the array because the base oxide was too thin. Later versions of the array, including the version used for all VCU samples measured in this work, were produced using thicker chips that were tuned to ensure acceptable sensing range for all antibodies.

**Table 2:** LOB, LOD, and LOQ Calculations for Each Cytokine, Procalcitonin, and CRP in a Simple EAB Matrix and Complex Serum Matrix^a^

Capture Ab	LOB dilution in EAB (pg/mL)	LOD dilution in EAB (pg/mL)	LOQ lowest real concentration with CV < 20% (pg/mL)	LOB dilution in serum (pg/mL)	LOD dilution in serum (pg/mL)	LOQ lowest real concentration in serum with CV < 20% (pg/mL)	Example of elevated level in disease (pg/mL)
IL6 400	0.6	1.4	5	7.1	13.0	25	>100 [^25^]
1L17A 400	0.5	0.9	5	6.5	23.3	*	>20 [^16^]
IL17F 400	6.5	18.2	50	56.2	127.6	*	46 [^26^]
IL-27 400	14.8	55.3	100	31.4	88.8	*	2000 [^27^]
IL-10 400	11.7	25.0	50	4.1	23.5	*	10–100 [ ^16^,^28^ ]
TNFa 800	13.0	22.4	50	273.7	581.1	*	4–200 [^25^]
PCT 400	34.3	102.1	250	2,114	2,911	*	>100 [^29^]
CRP 800	1,904	5,240	30,000	202,323	323,199	*	3 × 10^6^ [^30^]

a The complex serum matrix was prepared with a 1:5 dilution of pooled human serum from uninfected individuals in EAB with 30% FBS. The EAB matrix curves were prepared using the same target cocktail mixture, batch of StaphAIR chips, and analyzed on the same day. The complex serum replicates were evaluated on three different days, production batches, and preparations of target cocktail which resulted in larger standard deviations between replicates at each concentration; the coefficients of variation at most relevant concentrations were >20%, and LLOQ could not be defined*.

### Assay Specificity, Intra- and Interassay Precision, and Spike/Recovery

The specificity of the detection antibodies was verified by leaving one detection antibody out per array and measuring an expected decrease in signal for each matching target protein (Figure S8). Intra-assay specificity was evaluated by calculating the mean, standard deviation, and % CV for replicate spots on a single assay chip. For a single chip exposed to recombinant antigen cocktail in the complex serum matrix, the average intra-assay precision was <20% CV (Figure S9). Interassay precision was evaluated by applying 5 serum samples (3 infected and 2 control) from the VCU cohort to each production batch of StaphAIR arrays. All analyte responses were plotted batchwise as a density plot (Figure S10) which showed similar assay response profiles for all three batches. A one-way ANOVA was performed with assay response (Å) as the outcome and batch as a categorical factor. No significant differences between batches were observed (F­(2,510) = 1.48, p = 0.228), indicating that batch did not meaningfully influence the measurements. Spike recovery was assessed in complex serum matrix (sample 117 diluted 1:5 in EAB30). High, medium, and low concentrations of each cytokine were spiked into each sample. Each sample without any spiked cytokines (neat) was also evaluated. Recovered concentrations were calculated using linear fits to a calibration curve containing relevant concentrations. Percent recovery was calculated using a standard formula (eq 4).

4 $$\%\;\mathrm{recovery}=\frac{\mathrm{measured spiked sample}-\mathrm{measured unspiked sample}}{\mathrm{known spike concentration}}\times 100$$

IL-10 and IL-6 had acceptable average recovery over four serum samples (83% and 111% respectively), which is comparable to commercial Luminex assays (Figure S11). The other cytokines had low average % recovery. Since this assay was still in the developmental phase, more work was needed to develop a fully quantitative assay, but performance was sufficient for use as a semiquantitative assay (using thickness values rather than interpolated concentration values) for a proof-of-concept demonstration with the *S. aureus* infection and control serum samples from VCU.

### Dual-Scale StaphAIR Measurements of Serum Samples

Serum samples were evaluated in cohorts of 32–40 along with diluent-only control. Measurement date and production batch information were tracked throughout all assay and data analysis steps. Additionally, a full calibration curve with recombinant antigen targets in a complex serum matrix was performed for each production batch. All individual serum samples were diluted at a 1 in 5 ratio in EAB30 for incubation with the array overnight and processed with the amplification protocol the following morning. The 1:5 ratio was the highest concentration that enabled sufficient heterophilic blocking by FBS and was a compromise to enable all components of the array to function within an acceptable range. Seven images were collected for each sample (6, 11, 25, 50, 100, 250, and 500 ms exposures) by using a modified ZIVA AIR instrument purchased from Adarza Biosystems. The images were analyzed by using a desktop version of a custom version of Microvigene software created for Adarza Biosystems by VigeneTech. This software applied circles in a defined grid pattern to outline the areas of the array that were aggregated into the median CCD intensity. The software is integrated with a calibration formula for converting the intensity to thickness using calibration curves and camera parameters.

### Data Processing and Cleaning

All Microvigene output files were processed in RStudio and combined into a single data frame for further analysis. A total of 429 samples were evaluated in this study, of which data from 401 samples were included in the subsequent analysis. A total of 19 samples were eliminated from analysis due to high levels of assay interference, presumably due to a high load of heterophilic antibodies that was not mitigated by the inclusion of FBS in the sample diluent. These 19 samples were eliminated on the basis of the brightness of the anti-FITC probes on each array. The anti-FITC is a polyclonal goat antibody to which heterophilic antibodies bind. Samples were eliminated IF the optimal exposure time for the anti-FITC probes was lower than that used for most samples AND if the average thickness of all anti-FITC spots on those arrays was greater than a threshold value set by visually examining the array images. Samples that were eliminated by this metric had visibly bright anti-FITC spots bordering the array, and 74% of them were from individuals with *S. aureus* infection (Figure S12). Additionally, 9 samples were eliminated due to missing data for one or more probes on the array due to artifacts in the AIR image that prevented an accurate analysis. All thickness data were corrected by subtraction of the thickness of the blank area in the middle of the array to account for differences in base thickness between arrays.

### Statistical Analysis

Chi-squared tests for categorical variables and Mann–Whitney tests for numerical variables were used to compare the proportions or the means of characteristics between participants with or without *S. aureus* infection. *p* values were adjusted to account for multiple comparisons using false discovery rate.^31^ StaphAIR antibody measurements were assessed for their predictive potential to discriminate infected patients from control subjects using receiver operating characteristic (ROC) curve analysis, with overall predictive accuracy summarized by the area under the ROC curve (AUC).^32^ Antigen combinations were determined using best subsets selection with multivariate logistic regression models to identify groups of antibodies that best diagnosed *S. aureus* infection. Nonparametric estimates for the AUC, standard error, and 95% confidence intervals were computed for each predictor. Analyses were conducted using SAS version 9.4 and Python v3.8.5. All statistical tests were two sided, and a *p*-value of <0.05 was considered significant. Predictive machine learning model screening was performed using JMP Student edition version 18.2.2. K-fold cross validation was used (5-folds) to calculate mean and standard error for the testing data across folds. During each fold, the model was trained on 4 subsets and validated on the remaining subset. Default settings were used for all models. Sensitivity, specificity, and negative and positive predictive values (NPV, PPV) and likelihood ratios (LR+, LR−) were calculated for each model using standard formulas and true/false positives and negatives.^33^ The models did not return indeterminant results. R version 4.5 and OriginPro version 10.0.0.154 were used to create plots.

## Results and Discussion

A total of 401 serum samples were included in the formal analysis after removal of samples with missing data or assay interference. Characteristics of the study participants according to the infection status are presented in Table 3. Most participants were diagnosed with either a bone/joint infection or bacteremia, with a much smaller fraction suffering from lung infections or endocarditis. Individuals with infection were more likely to be male, identify as smokers, be younger, have lower albumin levels, and have a lower body mass index (BMI). Information about the infection severity and alternative diagnoses was not available.

**Table 3:** Characteristics of Study Participants According to Infection Status for the 401 Samples Included in the Analysis^a^

Variable	Control (*n* = 175)	Infection (*n* = 226)	*P* value
Infection Type (*n* (%))		Bacteremia: 90 (39.8)	
		Bone/Joint: 95 (42.0)	
		Endocarditis: 30 (13.3)	
		Lung: 11 (4.9)	
Outcome (*n* (%))		Adverse: 120 (53.1)	
		Infection controlled: 87 (38.5)	
		Unknown: 19 (8.4)	
Strain (*n* (%))		MRSA: 93 (41.2)	
		MSSA: 133 (58.8)	
Gender (*n* (%))	Female: 97 (55.4)	Female: 93 (41.2)	0.02
	Male: 78 (44.6)	Male: 133 (58.8)	
Age (mean (SD))	57.4 (12.6)	52.7 (16.0)	0.001
albumin (mean (SD))	4.3 (0.3)	3.0 (0.7)	2.0 × 10^–38^
BMI (mean (SD))	31.8 (7.9)	28.3 (7.0)	4.9 × 10^–7^
Smoker (*n* (%))	no: 145 (82.9)	no: 147 (66.2)	0.001
	yes: 30 (17.1)	yes: 75 (33.8)	
Race (*n* (%))	Asian: 1 (0.6)	Asian: 1 (0.5)	0.45
	Black: 72 (42.4)	Black: 109 (49.3)	
	Hispanic: 2 (1.2)	Hispanic: 5 (2.3)	
	White: 95 (55.9)	White: 106 (48.0)	
Hba1c (mean (SD))	6.0 (1.1)	6.5 (1.8)	0.07

a Samples from 429 patients were measured, but 28 were removed due to incomplete data or assay interference. Individuals with infection were more likely to be male, younger, identify as smokers, have lower albumin, and have lower body mass index (BMI)

A boxplot of analyte thickness in Ångstroms (Å), where thickness correlates with amount of material bound to the probe spot for each sample, is presented in Figure 2. Several cytokine and antibody analytes had significantly higher average thickness in the infection group compared to control. Notably, cytokines (IL-10, IL-6) and *S. aureus*-specific antibodies (CHIPS, Hla, IsdA, IsdB, LukS, and SelX) had *p* values <0.0001 representing important pathways of the inflammatory and adaptive immune response to *S. aureus*.

**Figure 2:**
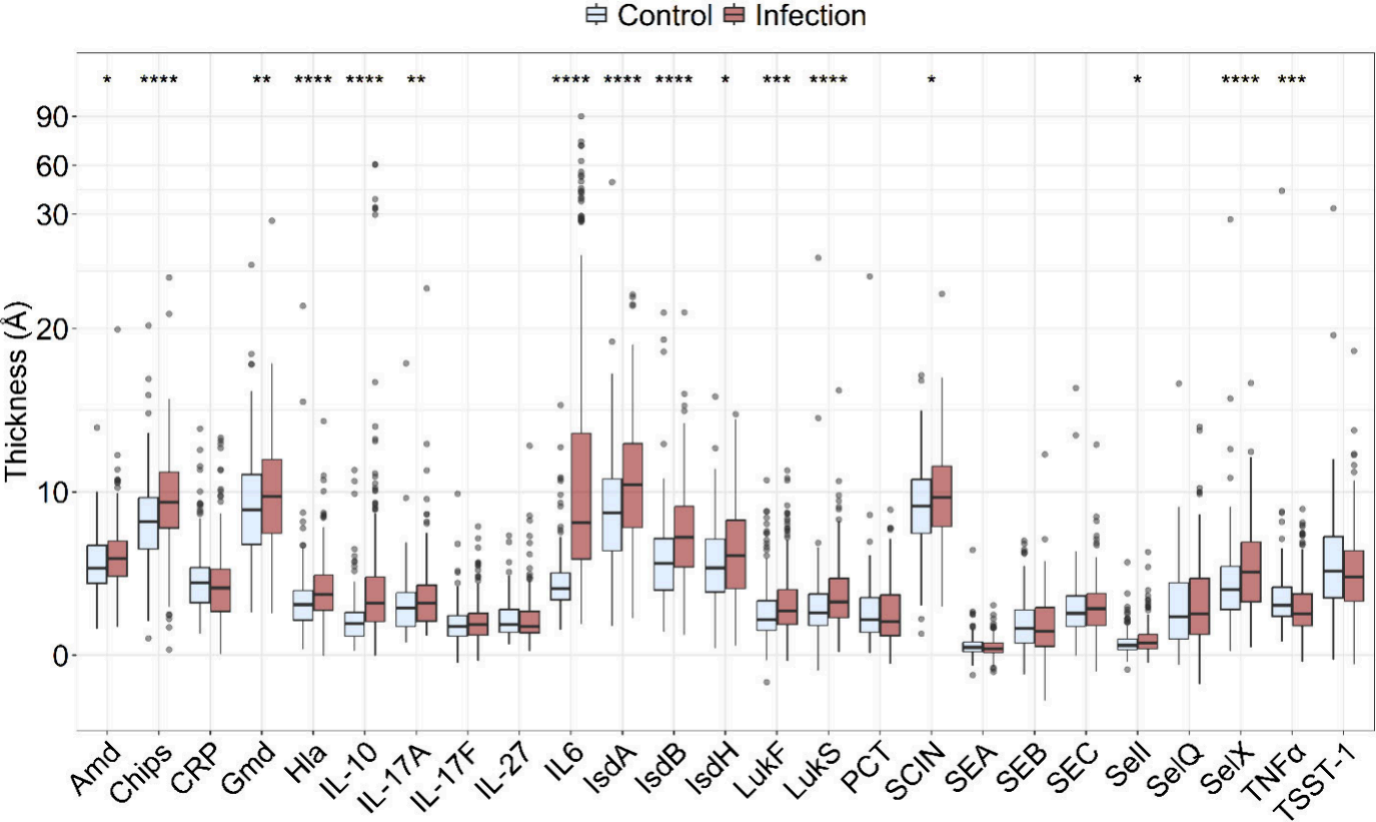
Boxplot comparing infection and control patients for all StaphAIR array predictors. Significance by Mann–Whitney U-test (*n* = 175 control, *n* = 226 infection, * *p* < 0.5, ** *p* < 0.01, *** *p* < 0.001, and **** *p* < 0.0001).

Univariate and multivariate logistic regression of all unscaled thickness data showed that IL-6 was the highest single contributor to the model for discriminating between infection and control groups (AUC 0.85) (Figure 3). All univariate contributions are presented in Figure S13. The addition of more variables improved the model performance, and the best combination of variables was IL-6, IL-10, IL-17A, TNFα, and IsdB (AUC 0.92). Several multivariate models had similarly high performance, and each was a combination of cytokines and antibodies against immunodominant *S. aureus* virulence factors (Figure S14).

**Figure 3:**
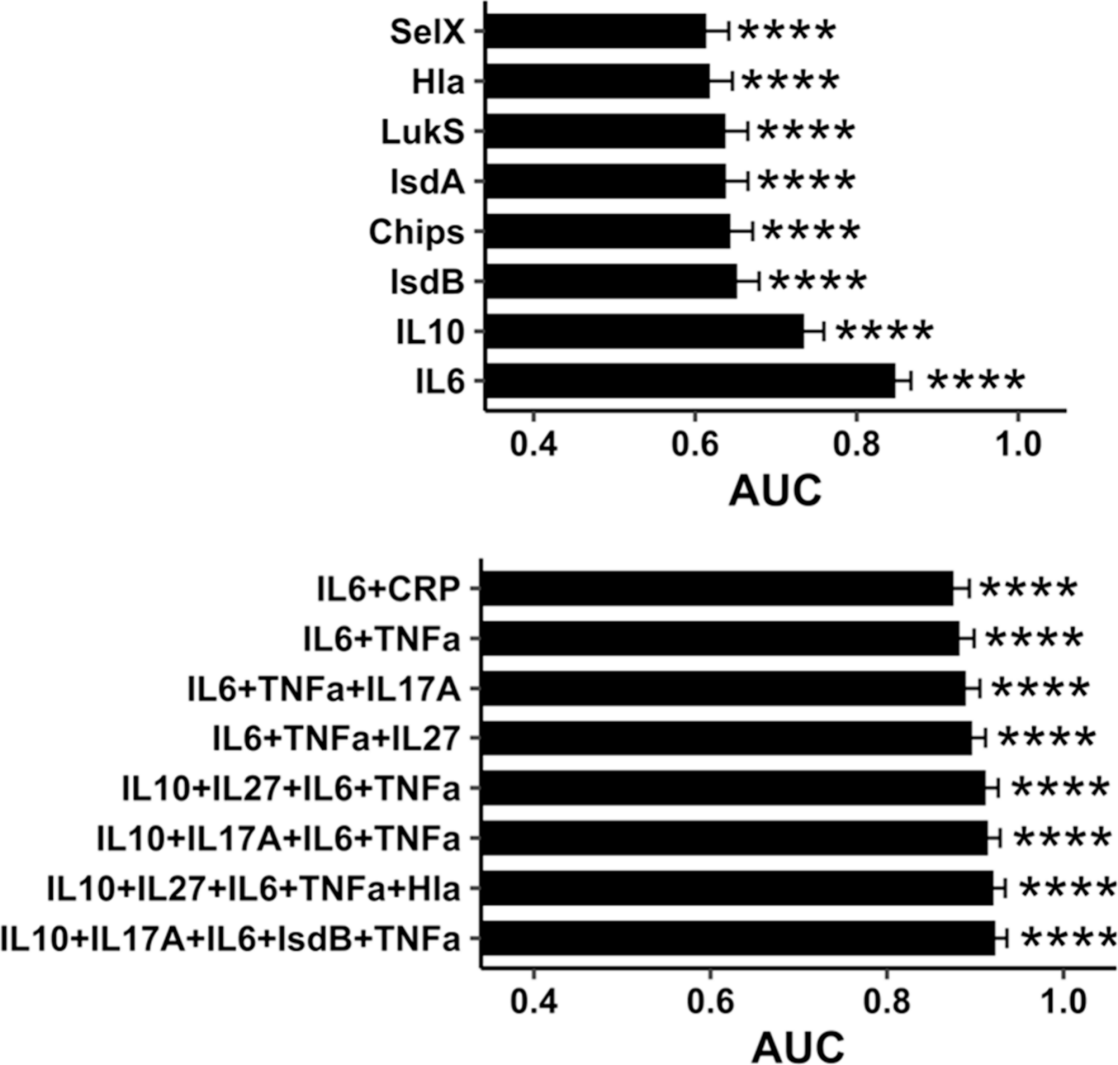
Highest-performing univariate and multivariate logistic regression models using StaphAIR thickness data from the 401 samples included in the analysis. Samples from 429 patients were measured, but 28 were removed due to incomplete data or assay interference. Data are depicted as mean ± SE with significance at * *p* < 0.05, ** *p* < 0.01, *** *p* < 0.001, and **** *p* < 0.0001.

The age, BMI, gender, albumin level, and smoker variables were assessed for confounding effects because they were significantly different between control and infection groups. Confounding variables affect both the independent and dependent variables.^34^ To assess potential confounding effects, each variable was added to the highest-performing multivariate logistic regression model, and the change in the value of the model coefficients was measured (Table 4). Albumin level was the only one that changed the regression coefficients by greater than 10%, indicating that it might impact the outcome,^34^ so it was considered along with the StaphAIR array analytes in a subsequent predictive model screen using 5-fold cross-validation. Supervised machine learning uses data with known outcomes to train a model to classify data into groups according to patterns in the predictive variables.^35^ This approach leverages complex multivariate data sets and identifies the predictive variables with the greatest influence on the outcome.

**Table 4:** Assessment of Potential Confounders Using Logistic Regression^a^

		logistic regression coefficient (% change)				
	AUC mean (95% CI)	IL-10	IL6	IL-17A	IsdB	TNFα
multivariate logistic regression	0.92 (0.88–0.95)	4.6	7.3	–2.3	0.7	–1.9
+ albumin	0.97 (0.96–0.99)	6.1 (32)	8.9 (21)	–3.7 (61)	1.1 (66)	–2.4 (31)
+ age	0.92 (0.89–0.95)	4.6 (5)	7.6 (4)	–2.2 (4.6)	0.6 (8.5)	–1.8 (1)
+ bmi	0.92 (0.90–0.94)	4.7 (3.2)	7.2 (1.2)	–2.3 (0.2)	0.6 (9.2)	–1.9 (1)
+ gender	0.92 (0.88–0.95)	4.8 (5.7)	7.4 (0.5)	–2.4 (5.1)	0.7 (2.2)	–1.9 (0.2)
+ smoker	0.91 (0.88–0.95)	4.6 (0.1)	7.1 (3.2)	–2.2 (2.1)	0.7 (2.2)	–1.7 (6.6)

a Each potential confounding variable was added to the highest performing 5-variable logistic regression model. The only variable that changed the regression coefficients by more than 10% was the albumin level.

The overall 5-fold cross-validated performance of the neural boosted, bootstrap forest, nominal logistic, boosted tree, and support vector machine models for predicting infection using the StaphAIR array information and albumin are presented in Table 5. In general, the addition of albumin information improved the performance of every model. The neural boosted model had the highest performance with (AUC 0.975) and without (AUC 0.914) albumin added to the 25 StaphAIR array predictive variables. The neural boosted model including albumin reached impressive positive and negative likelihood ratios (LR+: 15.65; LR–: 0.09). If this test were used in conjunction with a Fagan Nomogram^36^ with a patient who had a 50% pretest probability of disease, the post-test probability after a positive or negative test result would be 94% or 8.3% (Figure S15).

**Table 5:** Summary of Predictive Model Performance with and without Albumin in Validation Data^a^

Model		AUC	Sensitivity	Specificity	PPV	NPV	LRpos	LRneg
Neural Boosted		0.914 (0.895–0.934)	0.83 (0.74–0.92)	0.82 (0.71–0.92)	0.86 (0.80–0.92)	0.79 (0.69–0.89)	5.20 (2.66–7.74)	0.21 (0.11–0.30)
	+ albumin	0.975 (0.954–0.996)	0.91 (0.85–0.97)	0.92 (0.85–0.99)	0.95 (0.92–0.99)	0.86 (0.76–0.96)	15.65 (4.84–26.47)	0.09 (0.03–0.15)
Bootstrap Forest		0.900 (0.866–0.934)	0.81 (0.70–0.91)	0.77 (0.70–0.84)	0.82 (0.75–0.89)	0.76 (0.65–0.87)	3.65 (2.43–4.87)	0.25 (0.12–0.39)
	+ albumin	0.943 (0.927–0.960)	0.88 (0.85–0.91)	0.90 (0.83–0.97)	0.92 (0.88–0.96)	0.85 (0.79–0.91)	11.15 (3.99–18.31)	0.13 (0.09–0.17)
Nominal Logistic		0.890 (0.851–0.929)	0.80 (0.68–0.93)	0.81 (0.76–0.86)	0.85 (0.81–0.88)	0.77 (0.63–0.90)	4.34 (3.75–4.93)	0.24 (0.09–0.38)
	+ albumin	0.949 (0.919–0.979)	0.92 (0.84–0.99)	0.88 (0.78–0.97)	0.93 (0.89–0.97)	0.86 (0.73–0.98)	9.94 (2.70–17.18)	0.10 (0.01–0.18)
Boosted Tree		0.888 (0.850–0.926)	0.80 (0.68–0.92)	0.78 (0.70–0.86)	0.83 (0.76–0.89)	0.76 (0.64–0.88)	3.83 (3.00–4.66)	0.24 (0.11–0.38)
	+ albumin	0.915 (0.884–0.946)	0.85 (0.79–0.91)	0.85 (0.78–0.92)	0.88 (0.84–0.92)	0.81 (0.74–0.89)	6.37 (3.13–9.61)	0.18 (0.10–0.26)
Support Vector Machine		0.855 (0.842–0.867)	0.77 (0.67–0.87)	0.73 (0.61–0.84)	0.78 (0.70–0.87)	0.71 (0.62–0.80)	2.96 (2.18–3.75)	0.31 (0.22–0.41)
	+ albumin	0.953 (0.936–0.969)	0.89 (0.83–0.95)	0.88 (0.78–0.99)	0.93 (0.86–1.00)	0.83 (0.75–0.91)	7.08 (2.78–11.38)	0.12 (0.06–0.18)

a Mean AUC and 95% confidence intervals are reported for each model. Sensitivity, specificity, positive and negative predictive values (NPV, PPV), and likelihood ratios (LR) were calculated from the 5 folds and reported as mean and 95% CI.

The same model screen including albumin was performed for predicting adverse or controlled outcome of infection after one year, and the neural boosted model performed the best with AUC ± SE of 0.821 ± 0.026. The models were also assessed for predicting infection type between bone/joint or bacteremia, and the neural boosted model again had the highest performance (AUC 0.747 ± 0.029).

## Conclusions

Diagnosing deep chronic infections such as periprosthetic joint infection is challenging because of false-negative cultures and the occasional inability to detect pathogens. Although many biomarkers in serum have been identified with some predictive value, a combinatorial diagnostic that assesses markers of chronic inflammation and specific humoral immunity is required. To address this, we developed and validated an AIR approach that allows for quantitative assessments of pg/mL quantities of cytokines and mg/mL quantities of antigen-specific IgG in a single measurement and present proof-of-concept data demonstrating its utility for the intended purpose with a clinical cohort of 429 serum samples. The predictive models trained and cross-validated using this large set of *S. aureus* infection samples had good potential as diagnostic tools for *S. aureus* infection.

We found that measuring IL-6, IL-10, IL-17A, and TNFα plus antibodies against immunodominant *S. aureus* antigens (IsdB, alphatoxin/Hla, SelI, SelX, IsdH, IsdA, CHIPS, and LukF/S) produced the highest AUC in a predictive multivariate logistic regression model. The importance of these cytokines is consistent with the current model of a dual T_H_1/T_H_17 T-cell response to *S. aureus* infection.^37^ The neutrophil response at the early stages of infection is also critical to successfully clearing bacteria and preventing biofilm establishment.^38^ SelX is an inhibitor of neutrophil function as well as a superantigen capable of nonspecifically activating T-cells,^39^ so a robust response against that virulence factor is consistent with the current understanding of the coordinated innate and adaptive human immune response against *S. aureus* virulence factors.^11,40^ Additionally, the importance of IsdB/A/H, Hla, CHIPS, and LukF/S is consistent with previous work evaluating antibody biomarkers of *S. aureus* infection.^7,9,16^ The likelihood ratios from the top-performing neural boosted model that included albumin level as a predictive variable (LR+ 15.65, LR– 0.09) resulted in substantial increases in post-test probability of infection using a Fagan nomogram and outperform currently available ESR and CRP blood tests (LR+ 1.8–3.5 and LR– 0.3–0.8).^41^ The models did not perform as well at predicting the 1 year outcome of infection or discriminating between bacteremia and bone/joint infections.

The predictive models with the highest AUCs were the ones that evaluated diverse components of the immune response to *S. aureus*. For example, measuring cytokine readouts of the Th17 and Treg pathways (IL-6, IL-17A, and IL-10) together was more effective than measuring just one. This is consistent with work showing that measuring antibody responses against *S. aureus* virulence factors with diverse functions improved model performance for prediction of diabetic foot infection.^19^ Some of the most important single predictors of infection (IL-6, IL-10, IsdB, Chips, and IsdA) either do not appear in higher-order multivariate comparisons at all or do not appear together until 4- or 5- parameter comparisons. Some markers could be correlated and represent similar underlying biological functions, resulting in limited incremental value in multiparameter logistic regression until higher-order multivariate comparisons are evaluated. Similarly, multiparametric comparisons reach a point at which adding more predictors does not increase the predictive potential of the model, potentially due to redundancy in underlying biological function of markers. Additionally, some markers with poor univariate prediction seem to have value when added to multivariate models, which could indicate that they provide unique information not captured by more dominant markers. This seems to be the case for IL-27, CRP, and TNFa. These observations have important implications for diagnostic test development including the need to find a diverse yet compact group of biomarkers to simplify the test and subsequent analysis. The StaphAIR platform combined with regression analysis could function as a multiplex screening tool to identify the most relevant predictive biomarkers for an outcome variable before developing a final assay (potentially using tools already in clinical laboratories) for clinical or point-of-care use.

The cytokine response probably had high discriminatory value in this sample set because the samples were all culture-positive *S. aureus* infections, which masked any higher discriminatory potential of *S. aureus*-specific antibodies. To fully evaluate the importance of *S. aureus*-specific antibody detection in identifying *S. aureus* infection, future work will include samples from patients with bone and joint infections caused by *S. aureus*, *Streptococcus agalactiae*, *Cutibacterium acnes*, and other species of bacteria. If the models were trained with data from patients with infections due to a more diverse set of pathogens, then the *S. aureus*-specific antibodies would probably be more important predictive variables for identifying infections caused by *S. aureus*. Similarly, some of the cytokines with seemingly redundant predictive utility in this study may have a more complementary function in the context of other infections or diseases.

The ability to simultaneously detect cytokines and *S. aureus*-specific antibodies is a technical leap that distinguishes StaphAIR from other immunoassay techniques. This is the first immunoassay that has the versatility to measure 1-million-fold differences in concentration of mixed-function analytes like immunoglobulins and inflammatory proteins with a single sample dilution. More work is needed to translate this proof-of-concept study from the laboratory to the clinic, but if that hurdle is surmounted, then this dual-scale approach could offer simpler sample processing by eliminating the need for splitting the sample for multiple assays. It could offer a rapid and cohesive readout as a companion tool for gold-standard microbial culture and could be a useful research tool for monitoring immune response during vaccine development and clinical trials. For example, undetectable levels of IL-2 and IL-17A on both the day of vaccination with the failed Merck V710 *S. aureus* vaccine and the day of hospitalization were associated with increased mortality in patients who received the vaccine,^42^ which highlights the importance of longitudinal tracking of IL-17A and other cytokines. Additional applications include prescreening patients for elective surgery to assess known protective or pathogenic adaptive immune phenotypes, such as the Isd/Atl antibody ratio.^43^ The ability to track the cytokine response over time could provide valuable information about the T-cell response and may be able to predict chronic infection. Further expansion of the array to detect immune checkpoint inhibitors could enhance the utility of the assay for understanding the causes of chronic infection and tracking that condition over time.^44^

## Supplementary Material



## Notes

Detailed information regarding antibodies used in the array, probe molecule formulations and array layout, protocol for use of low-volume consumables, cross-reactivity assessment, dilution curves, assay performance calculations, univariate logistic regression analysis, multivariate logistic regression models, and Fagan nomogram calculations (PDF)
