# Supplementary material for: Dual-Scale StaphAIR: Predictive Modeling for the Diagnosis of S. aureus Infection via Simultaneous Detection and Quantification of Cytokines and Antibodies
Source: Anal Chem. 2026 May 16;98(21):15599–608. doi: 10.1021/acs.analchem.6c00764 (PMC13234818; doi:10.1021/acs.analchem.6c00764)
Supplement: Supplementary file 1 [file ac6c00764_si_001.pdf]

Supporting Information

Dual-scale StaphAIR: Predictive modeling for the diagnosis of *S. aureus* infection via simultaneous detection and quantification of cytokines and antibodies

Alanna M. Klose[1], Christopher A. Beck[2,3], Stephen L. Kates[4], Gowrishankar Muthukrishnan[2], Edward M. Schwarz[2], Benjamin L. Miller[1*]

1. Department of Dermatology, University of Rochester, Rochester, New York 14627, USA,

2. Center for Musculoskeletal Research, University of Rochester Medical Center, Rochester, New York 14627, USA.

3. Department of Biostatistics and Computational Biology, University of Rochester Medical Center, Rochester, New York 14627, USA.

4. Department of Orthopaedic Surgery, Virginia Commonwealth University, Richmond, Virginia 23284, USA.

Corresponding Author: Benjamin L. Miller University of Rochester, Rochester, New York 14627, USA. benjamin_miller@urmc.rochester.edu. Phone: (585) 275-9805

Table of Contents

Table S1 The capture and detection antibodies used for the dual-scale StaphAIR array. S3

Table S2 Formulations for each ligand in the dual scale StaphAIR array. S4

Figure S1 Array layout for all ligands covalently attached to the chip in the dual-scale StaphAIR array. S5

Figure S2 Printing, blocking, and loading chips into low volume consumables.. ....S6

Figure S3 Incubation of arrays in SA-poly-HRP and DAB, washing, and drying. S7

Figure S4 Images of arrays incubated in serum samples diluted EAB alone versus EAB plus 30% FBS S8

Figure S5 Dilution curves of cytokines, procalcitonin, and CRP in EAB matrix S9

Figure S6 Dilution curves of cytokines, procalcitonin, and CRP in complex serum matrix. S10

Figure S7 Serial dilutions of pooled positive human serum. S11

Figure S8 Cross-reactivity assessment (n-1 detection antibodies). S11

Figure S9 Assessment of intra-assay precision. S12

Figure S10 Assessment of batch effects and inter-assay precision. S13

Figure S11 Concentration spike recovery on StaphAIR array in complex serum matrix. S14

Figure S12 Example of Risk Adjust samples eliminated due to heterophilic antibody assay interference. S15

Figure S13 Full univariate logistic regression analysis using StaphAIR thickness data from cleaned data (401 serum samples). S16

Figure S14 Top 8 multivariate logistic regression models for combinations of 2, 3, 4, and 5 predictive variables.. ....S17

Figure S15 Fagan nomogram calculation of post-test probability with pre-test probability of 50%..S18

Table S1 Table S1 The capture and detection antibodies used for the dual-scale StaphAIR array. Capture antibodies were covalently attached to the AIR chip surface. Detection antibodies were biotin-conjugated to enable the amplification process.

Antibody	Sandwich assay Role	Company	Catalog Number	Type and Species
anti-human/mouse IL-27	Capture	Biolegend	516901	monoclonal mouse
anti-human IL-6	Capture	Biolegend	501125	monoclonal rat
anti-human IL-17A	Capture	Biolegend	512702	monoclonal mouse
anti-human primate IL-17F	Capture	R&D Systems	AF1335-SP	polyclonal goat
anti-human TNFalpha	Capture	Peprotech	500-M26	mouse monoclonal
anti-human IL-10	Capture	Biolegend	506802	monoclonal rat
anti-human CRP ms IgG2B	Capture	R&D Systems	MAB17071	mouse monoclonal
anti-human procalcitonin	Capture	R&D Systems	MAB83502-100	monoclonal mouse
biotin anti-human IL-27	Detection	R&D Systems	BAF2526	polyclonal goat
biotin anti-human primate IL-17F	Detection	R&D Systems	BAF1335	polyclonal goat
biotin-anti-human procalcitonin	Detection	Novus Biologicals	NBP3-00233B	polyclonal rabbit
bioin-anti-human IL-10	Detection	Biolegend	501502	monoclonal rat
biotin anti-human IL-17A	Detection	Biolegend	518902	polyclonal goat
biotin anti-human TNFalpha	Detection	Biolegend	502903	mouse monoclonal
biotin anti-human IL-6	Detection	R&D Systems	BAF206	polyclonal goat
anti-human CRP polyclonal	Detection	R&D Systems	AF1707	polyclonal sheep

Table S2 Formulations for each ligand in the dual scale StaphAIR array.

	# drops	Probe	Stock Concentration (µg/mL)	Working dilution (µg/mL)	printed conc. (µg/mL)	volume of probe (µL)	volume of 7.4 pH PBS (µL)	volume of 25% trehalose (µL)
1	1	anti-FITC dia	1596		500	3.1	6.1	0.8
2	1	human IgG	5000		800	1.6	7.6	0.8
3	1	IL-10 mAb Bioleg. 506802 dialyzed	602		400	6.6	2.6	0.8
4	1	IL-17A mAb Bioleg. dialyzed	500		400	8.0	1.2	0.8
5	1	IL-17F pAb R&D AF1335	633		400	5.7	2.5	0.8
6	1	IL-6 mAb Bioleg.	2000		400	2.0	7.2	0.8
7	1	TNFα mAb Peprotech	1000		800	8.0	1.2	0.8
8	1	IL-27 mAb BL dialyzed	585		400	6.8	2.4	0.8
9	1	Procalcitonin R&D 83502	864		795	9.2	0.0	0.8
10	1	CRP mAb R&D IgG2B	923		780	8.5	0.7	0.8
11	2	SEA	951		600	6.3	2.9	0.8
12	2	SEB	954		600	6.3	2.9	0.8
13	2	SEC	951		600	6.3	2.9	0.8
14	1	TSST-1	1306		800	6.1	3.1	0.8
15	1	SelQ	500		460	9.2	0.0	0.8
16	2	Sel I	487		400	8.2	1.0	0.8
17	1	Sel X	1814		500	2.8	6.4	0.8
18	1	alpha toxin	1400		600	4.3	4.9	0.8
19	1	IsdB dia 1:4	1827	456.75	80	1.8	6.6	1.6
20	1	IsdH dia 1:10	3376	337.6	100	3.0	6.2	0.8
21	1	Gmd dia 1:20	12928	646.4	150	2.3	6.1	1.6
22	1	SCIN dia 1:20	9725	486.25	100	2.1	7.1	0.8
23	1	IsdA dia 1:5	2500	500	100	2.0	7.2	0.8
24	1	Amd dia 1:20	10600	530	100	1.9	7.3	0.8
1	1	Chips dia 1:10	4059	405.9	100	2.5	6.7	0.8
2	1	LukS 1:2	583		300	4.6	3.6	0.8
3	1	LukF	1624		300	1.8	7.4	0.8

hIgG	FITC	FITC	FITC	FITC	FITC	FITC	FITC	FITC	FITC	FITC	FITC	FITC	hIgG
FITC	Blank	Blank	Blank	Blank	Blank	Blank	FITC	Blank	Blank	Blank	Blank	Blank	Blank	FITC
FITC	Blank	Blank	Blank	Blank	Blank	Blank	FITC	Blank	Blank	Blank	Blank	Blank	Blank	FITC
FITC	IL-10 400	IL-10 400	IL-10 400	IL-10 400	IL-10 400	IL-10 400	FITC	IL-17A 400	IL-17A 400	IL-17A 400	IL-17A 400	IL-17A 400	IL-17A 400	FITC
FITC	Il-17F pAb 400	Il-17F pAb 400	Il-17F pAb 400	Il-17F pAb 400	Il-17F pAb 400	Il-17F pAb 400	FITC	IL-6 400	IL-6 400	IL-6 400	IL-6 400	IL-6 400	IL-6 400	FITC
FITC	TNFa Pep 800	TNFa Pep 800	TNFa Pep 800	TNFa Pep 800	TNFa Pep 800	TNFa Pep 800	FITC	Il-27 mAb 400	Il-27 mAb 400	Il-27 mAb 400	Il-27 mAb 400	Il-27 mAb 400	Il-27 mAb 400	FITC
FITC	PCT 800	PCT 800	PCT 800	PCT 800	PCT 800	PCT 800	FITC	CRP 800	CRP 800	CRP 800	CRP 800	CRP 800	CRP 800	FITC
FITC	SEA	SEA	SEA	SEA	SEA	SEA	FITC	SEB	SEB	SEB	SEB	SEB	SEB	FITC
FITC	SEC	SEC	SEC	SEC	SEC	SEC	FITC	TSST-1	TSST-1	TSST-1	TSST-1	TSST-1	TSST-1	FITC
FITC	Blank	Blank	Blank	Blank	Blank	Blank	FITC	SelQ	SelQ	SelQ	SelQ	SelQ	SelQ	FITC
FITC	SelI	SelI	SelI	SelI	SelI	SelI	FITC	SelX	SelX	SelX	SelX	SelX	SelX	FITC
FITC	alpha-toxin	alpha-toxin	alpha-toxin	alpha-toxin	alpha-toxin	alpha-toxin	FITC	IsdB	IsdB	IsdB	IsdB	IsdB	IsdB	FITC
FITC	IsdH	IsdH	IsdH	IsdH	IsdH	IsdH	FITC	Gmd	Gmd	Gmd	Gmd	Gmd	Gmd	FITC
FITC	SCIN	SCIN	SCIN	SCIN	SCIN	SCIN	FITC	IsdA	IsdA	IsdA	IsdA	IsdA	IsdA	FITC
FITC	Amd	Amd	Amd	Amd	Amd	Amd	FITC	Chips	Chips	Chips	Chips	Chips	Chips	FITC
FITC	LukS	LukS	LukS	LukS	LukS	LukS	FITC	LukF	LukF	LukF	LukF	LukF	LukF	FITC
hIgG	FITC	FITC	FITC	FITC	FITC	FITC	FITC	FITC	FITC	FITC	FITC	FITC	FITC	hIgG

Figure S1 Array layout for all ligands covalently attached to the chip in the dual-scale StaphAIR array.

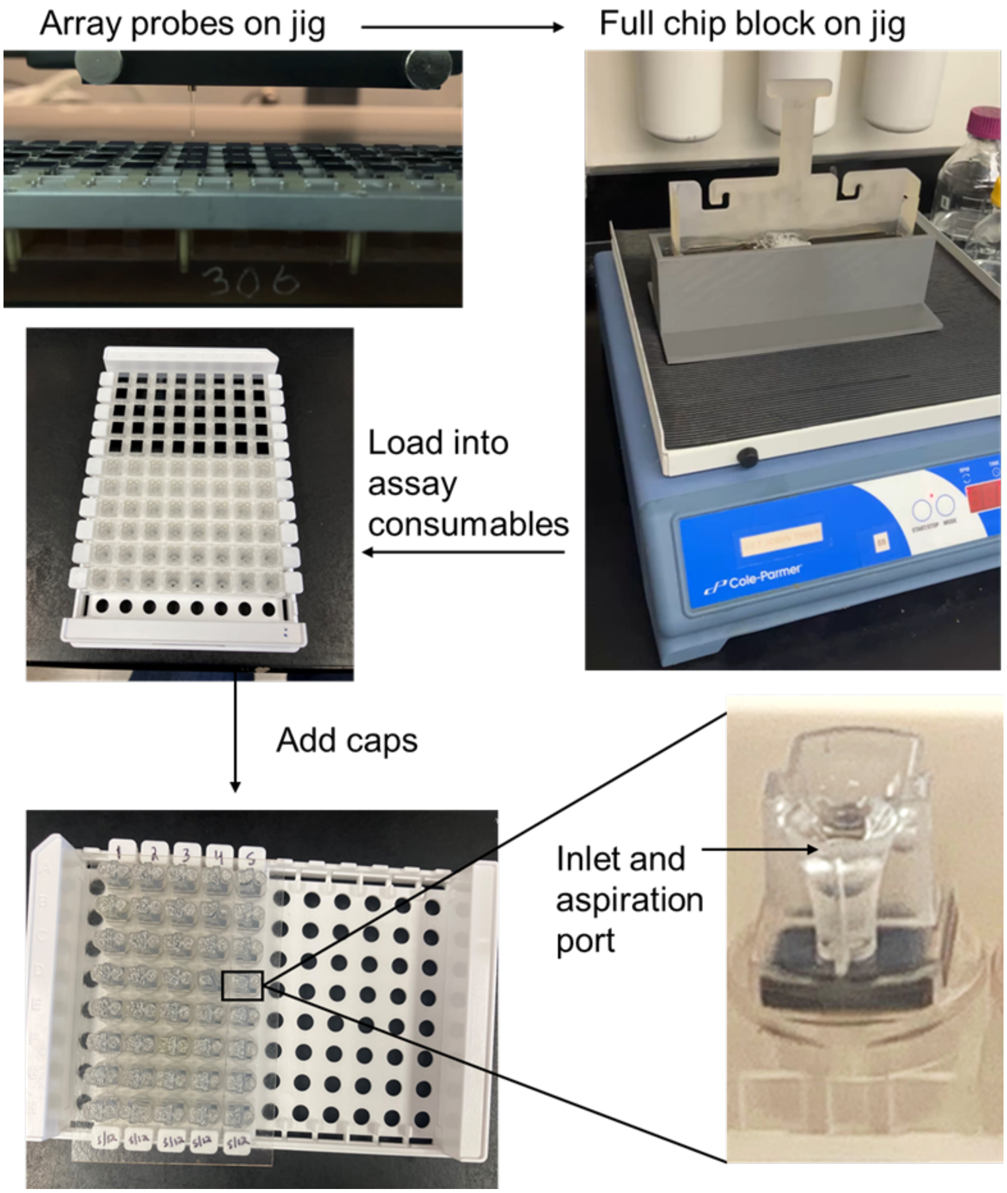

Figure S2 Printing, blocking, and loading chips into low volume consumables. Changes to array production to enable assays in low volume ZIVA consumables included printing, blocking, and stabilizing chips on jigs before packaging into consumables that snapped into a custom 96-well plate.

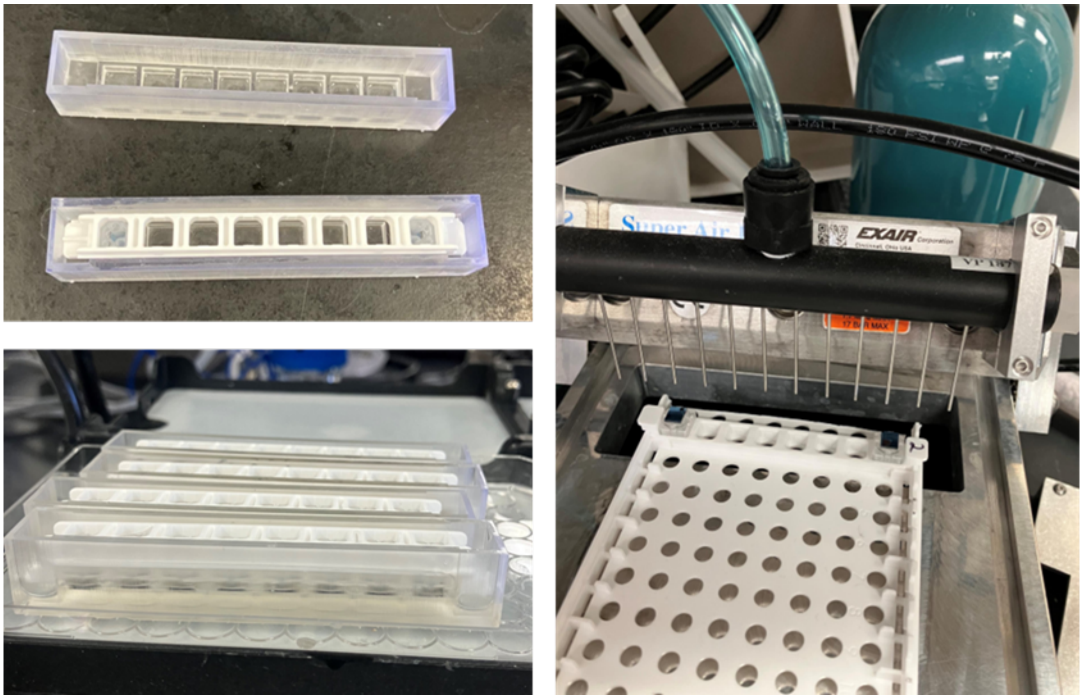

Figure S3 Incubation of arrays in SA-poly-HRP and DAB, washing, and drying. All steps after the dAb incubation were performed in the 3D-printed resin wells on the left. The final wash in Nanopure water and dry in a stream of nitrogen gas is performed using the custom-built tool shown on the right.

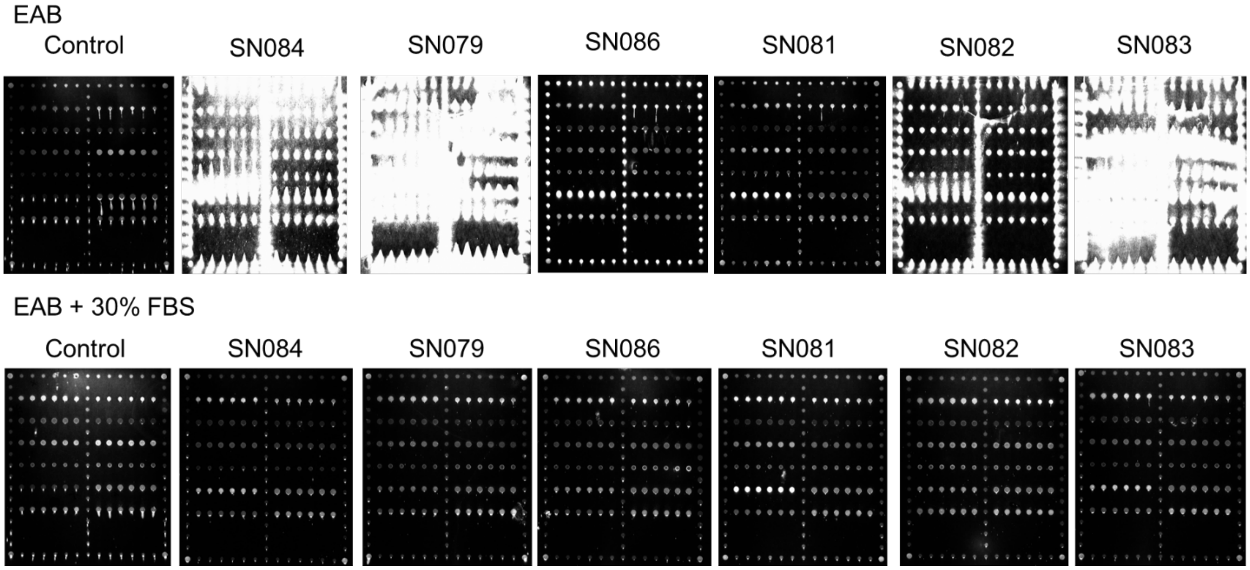

Figure S4 Images of arrays incubated in serum samples diluted EAB alone versus EAB plus 30% FBS. The addition of FBS is an effective block against assay interference suspected to be due to heterophilic antibodies in these serum samples.

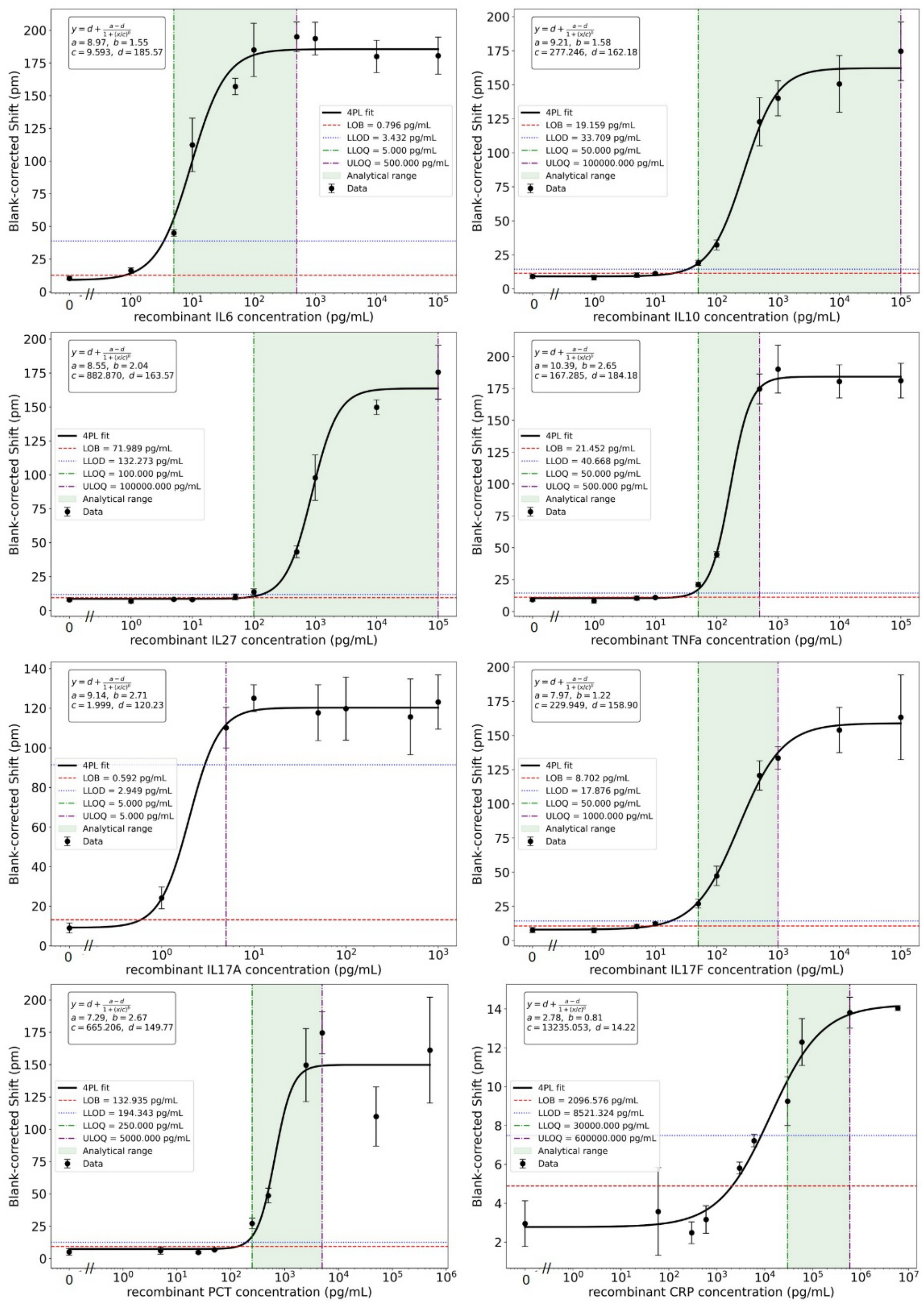

Figure S5 Dilution curves of cytokines, procalcitonin, and CRP in EAB matrix. A 4-parameter logistic fit was used to calculate the limit of the blank (LOB), lower limit of detection (LLOD), and lower and upper limits of quantitation (LLOQ and ULOQ). Error bars represent measurements on 3 replicate arrays (3 separate chips for each measurement). The LLOQ was defined as the lowest real concentration measured with a coefficient of variation (CV) of less than 20%. The ULOQ was defined as the highest concentration with CV < 20% and a mean signal not within 5% of the upper asymptote of the fitted 4PL curve.

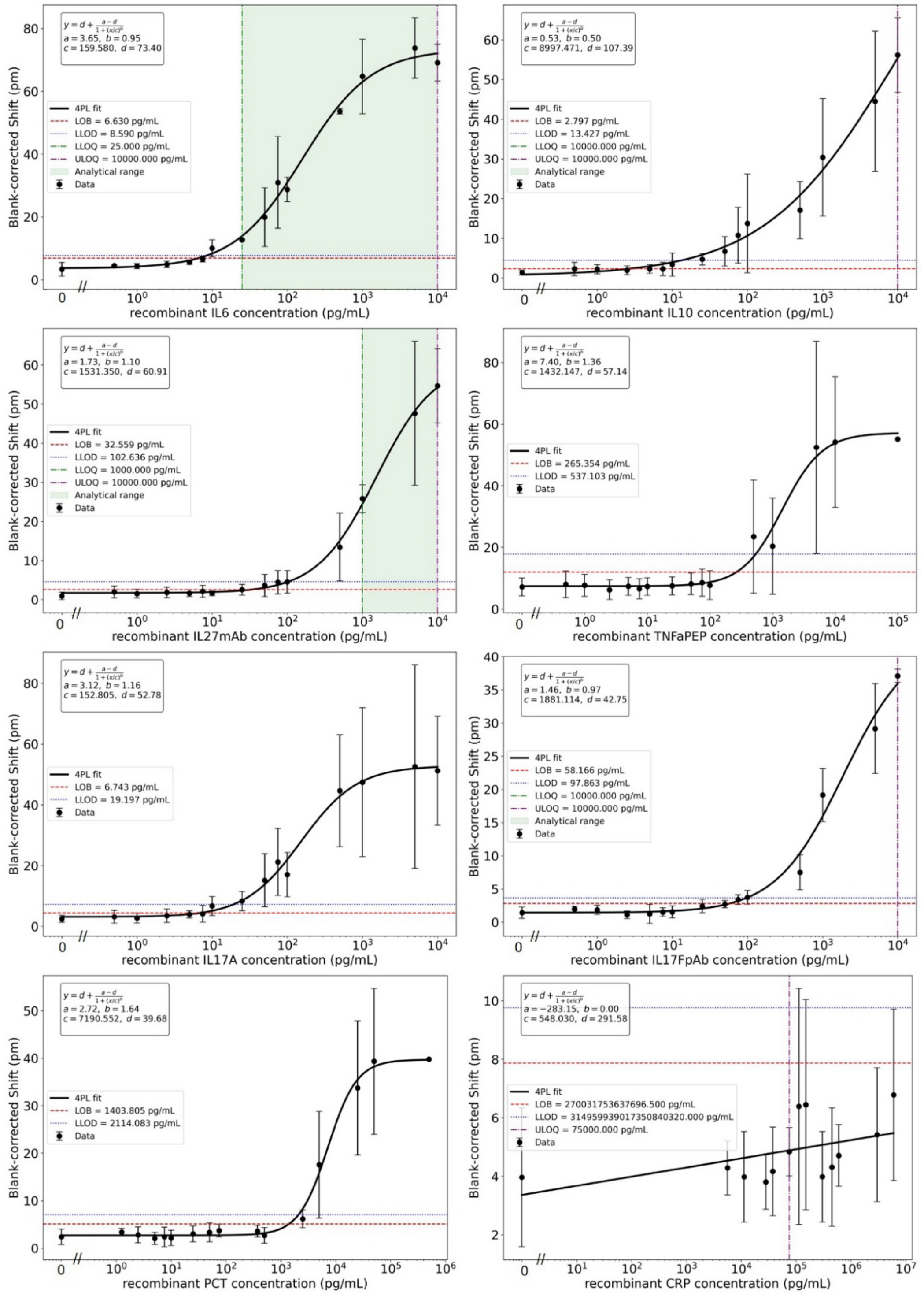

Figure S6 Dilution curves of cytokines, procalcitonin, and CRP in a complex serum matrix. The matrix consisted of a 1:5 dilution of pooled human serum from uninfected individuals in EAB30 (EAB containing 30% FBS). The error bars represent measurements on 3 replicate arrays (3 separate chips for each measurement) analyzed on three different days, production batches of StaphAIR chips, and preparations of spiked target cocktail.

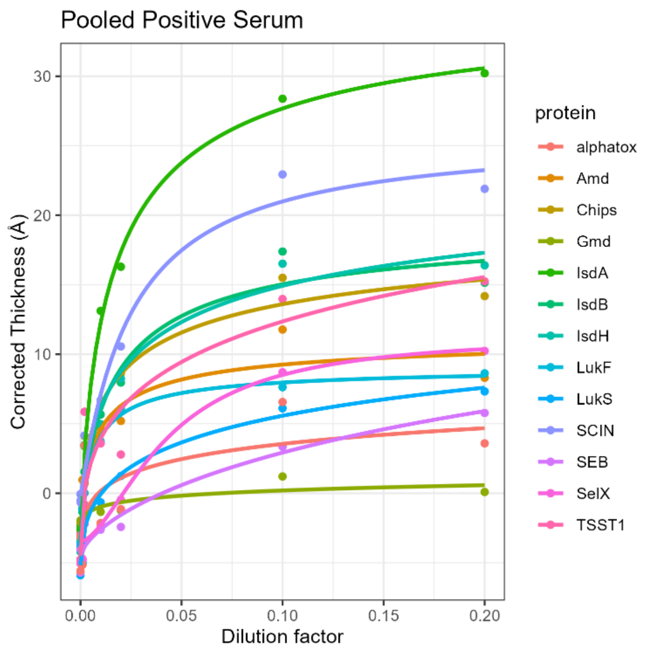

Figure S7 Serial dilutions of pooled positive human serum. Pooled serum from individuals with a culture-confirmed *S. aureus* infection was serially diluted and incubated with a StaphAIR array. As expected, the antibody response in these samples increases with dilution factor.

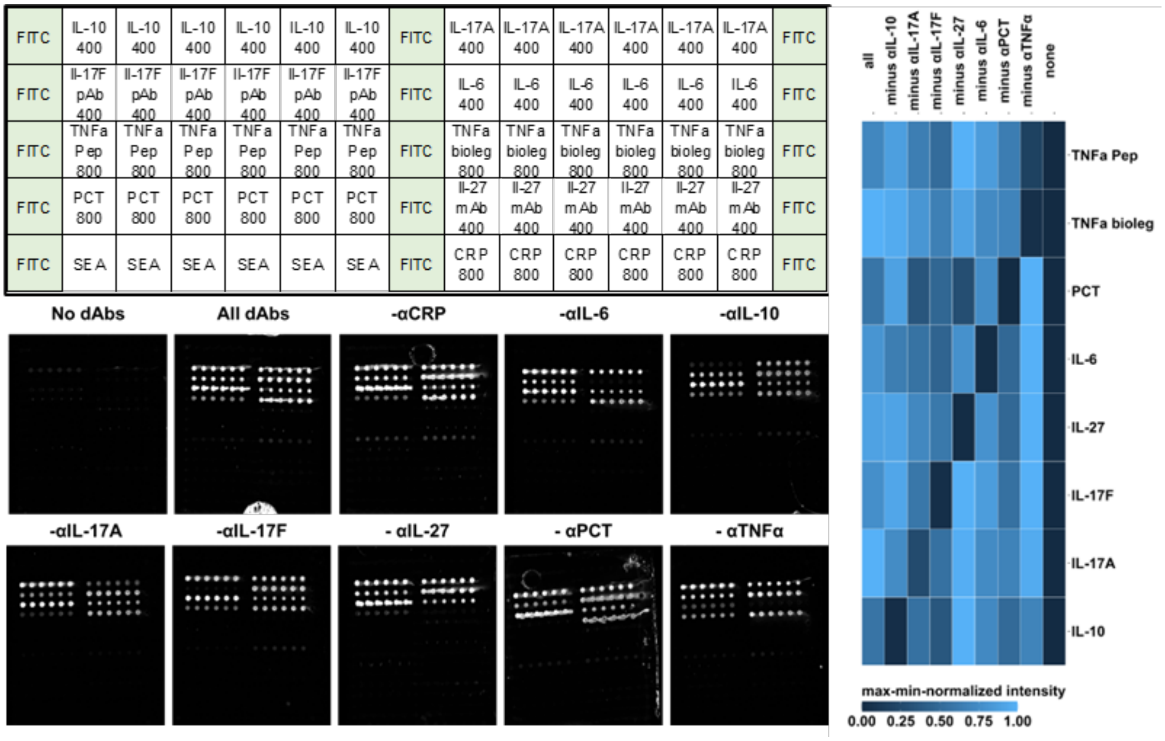

Figure S8 Cross-reactivity assessment (n-1 detection antibodies). All arrays were incubated with 10 ng/mL of cytokines and PCT, and 1 µg/mL of CRP. One detection antibody was left out of each condition. The detection antibodies have good specificity for their cytokine targets.

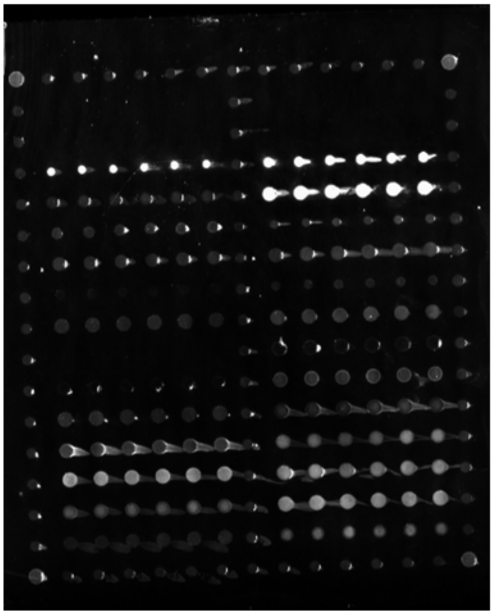

protein	mean (Å)	SD	%CV	N replicates
Amd	5.8	0.3	5.3	6
CRP	6.5	0.9	13.4	6
Chips	12.4	0.6	4.9	6
Gmd	7.2	1.2	16.4	6
IL10	7.4	1.5	19.7	6
IL17A	10.1	1.1	11.3	6
IL17F	3.9	0.3	7.3	6
IL27	1.9	0.4	23.0	6
IL6	26.1	1.3	5.0	6
IsdA	10.5	0.8	7.3	6
IsdB	4.3	1.0	23.2	6
IsdH	6.6	0.6	9.7	6
LukF	4.5	0.5	11.9	6
LukS	3.2	0.6	17.3	6
PCT	5.0	0.6	12.7	6
SCIN	12.8	0.6	5.0	6
SEA	0.8	0.2	30.4	6
SEB	1.0	0.5	44.9	6
SEC	4.3	0.6	14.6	6
SelI	0.7	0.3	45.6	6
SelQ	1.9	0.3	15.1	6
SelX	5.8	0.8	13.4	6
TNFa	3.5	1.3	38.1	6
TSST1	6.3	0.6	10.0	6
alphatox	4.2	0.9	20.5	6
average			17.0	

Figure S9 Assessment of intra-assay precision. This is a 100 ms exposure image of D7-1 dilution condition in complex serum matrix (EAB30 with 1:5 dilution of a pooled serum sample). Quantification was performed with optimal exposure time for each analyte to avoid saturation of response. The average % CV was <20% including all probes on the chip.

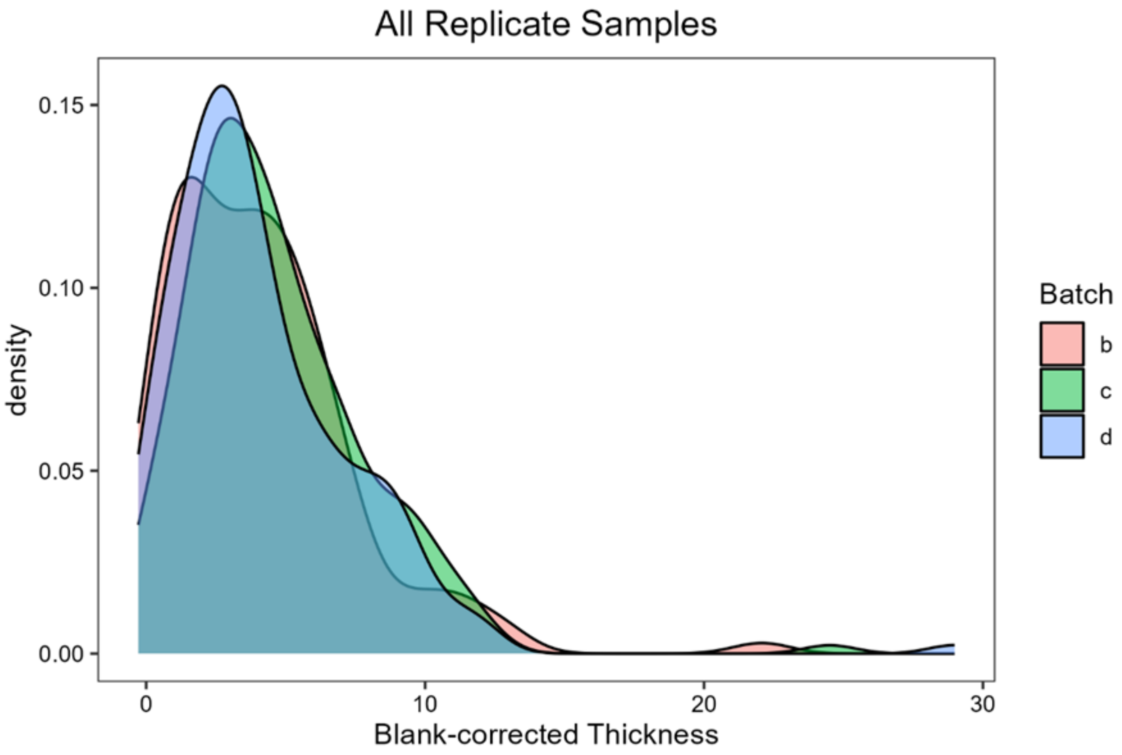

Figure S10 Assessment of batch effects and inter-assay precision. Inter-assay precision was evaluated by applying 5 serum samples (3 infected and 2 control) from the VCU cohort to each production batch of StaphAIR arrays. All analyte responses were plotted batchwise as a density plot which showed similar assay response profiles for all three batches. A one-way ANOVA was performed with assay response (Å) as the outcome and batch as a categorical factor. No significant differences between batches were observed ($F(2,510) = 1.48$, $p = 0.228$), indicating that batch did not meaningfully influence the measurements.

	IL10	IL17A	IL17F	IL27	IL6	TNFa
115-hi	-10%	44%	62%	23%	59%	43%
115-lo	197%	-1%	40%	-2%	161%	35%
115-mi	244%	3%	107%	-1%	178%	58%
115-neat						
116-hi	97%	39%	57%	11%	106%	88%
116-lo	130%	146%	25%	31%	145%	59%
116-mi	194%	83%	82%	21%	176%	66%
116-neat						
117-hi	74%	86%	87%	9%	52%	49%
117-lo	57%	112%	37%	-5%	37%	20%
117-mi	90%	61%	43%	36%	33%	12%
117-neat						
118-hi	28%	32%	89%	15%	163%	66%
118-lo	-87%	-210%	20%	-50%	97%	-5%
118-mi	-21%	43%	96%	39%	129%	24%
118-neat						
**average**	83%	37%	62%	11%	111%	43%
**reported luminex values**	90%	72%	75%	--	78%	71%

Figure S11 Concentration spike recovery on StaphAIR array in complex serum matrix. The serum matrix was composed of individual serum sample 117 diluted 1:5 in EAB30. The highlighted boxes are those with recovery between 80-120%. Reported Luminex values are from the human XL cytokine Luminex® Kit Performance Assay (R&D Systems, Catalog # FCSTM18B) and the human High Sensitivity Cytokine B Premixed Magnetic Luminex® Performance Assay (R&D Systems, | Catalog # FCSTM14).

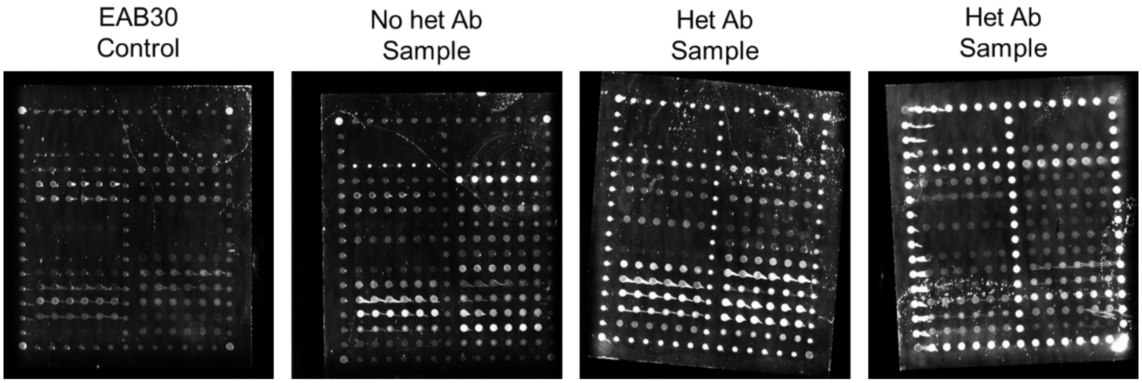

**Infection Status**	**N**	**%**
Infected	14	74
Control	5	26
**Type of infection**	**N**	**%**
bone/joint	7	50%
bacteremia	3	21%
endocarditis	3	21%
lung	1	7%
**Infection Outcome**	**N**	**%**
adverse outcome	8	57%
infection controlled	4	29%
unknown	2	14%

Figure S12 Example of Risk Adjust samples eliminated due to heterophilic antibody assay interference. The αFITC spots around the outside of the array are polyclonal goat antibodies and increase in reflectivity when there is assay interference due to heterophilic antibodies in the human serum sample. 74% of these samples were from individuals with *S. aureus* infection.

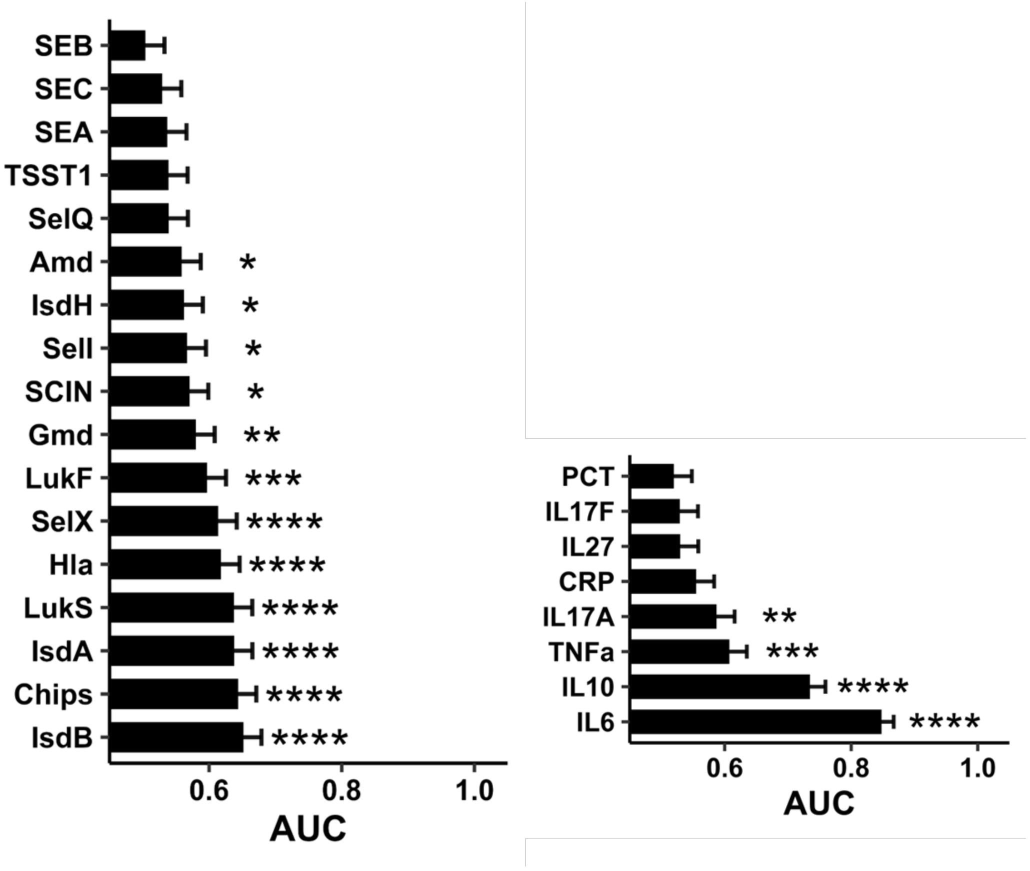

Figure S13 Full univariate logistic regression analysis using StaphAIR thickness data from cleaned data (401 serum samples). Data are depicted as mean +/– SE with significance at * p < 0.05 ** *p* < 0.01*** *p* < 0.001 and **** *p* < 0.0001.

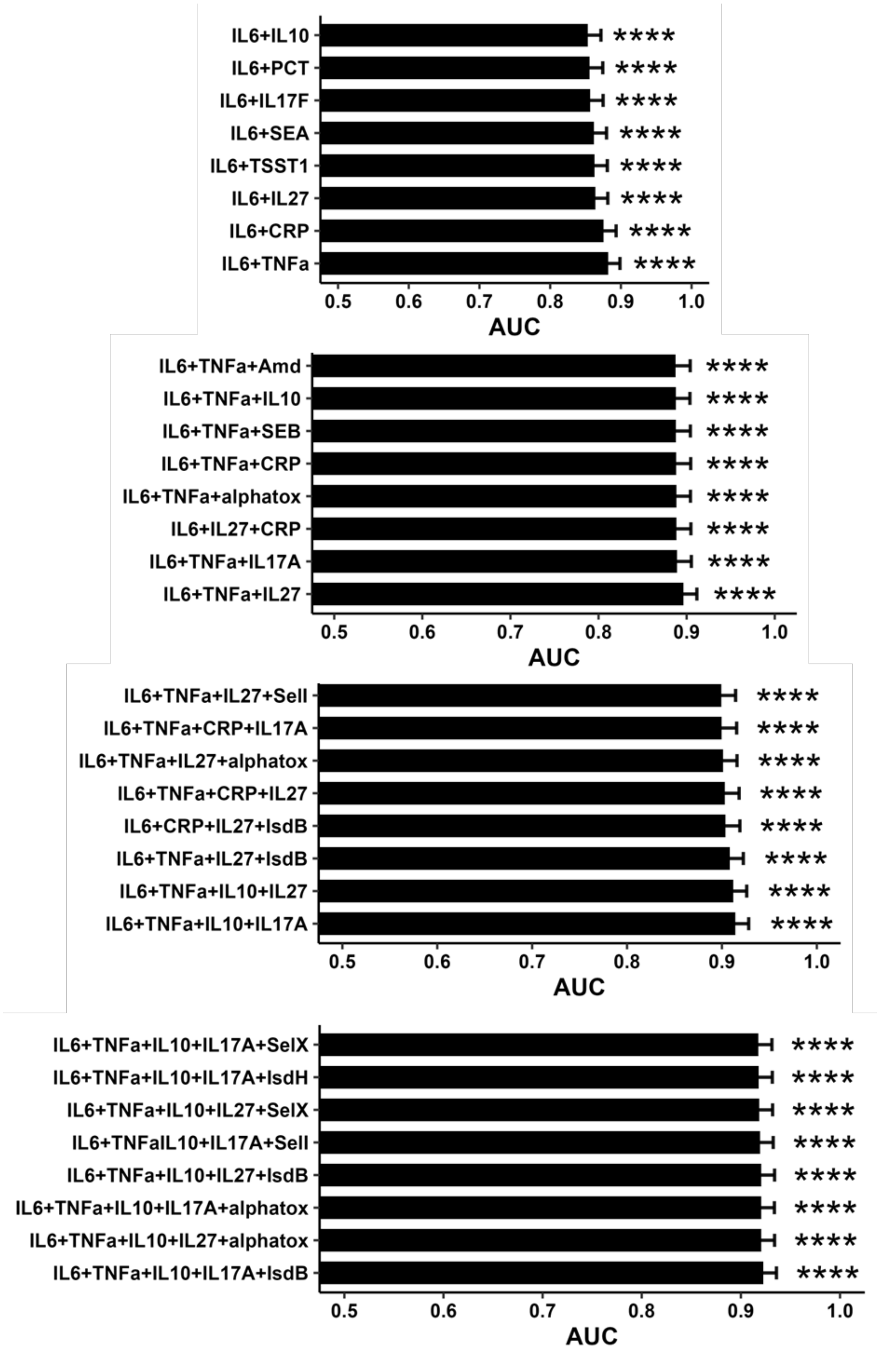

Figure S14 Top 8 multivariate logistic regression models for combinations of 2, 3, 4, and 5 predictive variables. Analysis was performed using StaphAIR thickness data from all 401 samples. Data are depicted as mean +/– SE with significance at * $p < 0.05$ ** $p < 0.01$ *** $p < 0.001$ and **** $p < 0.0001$.

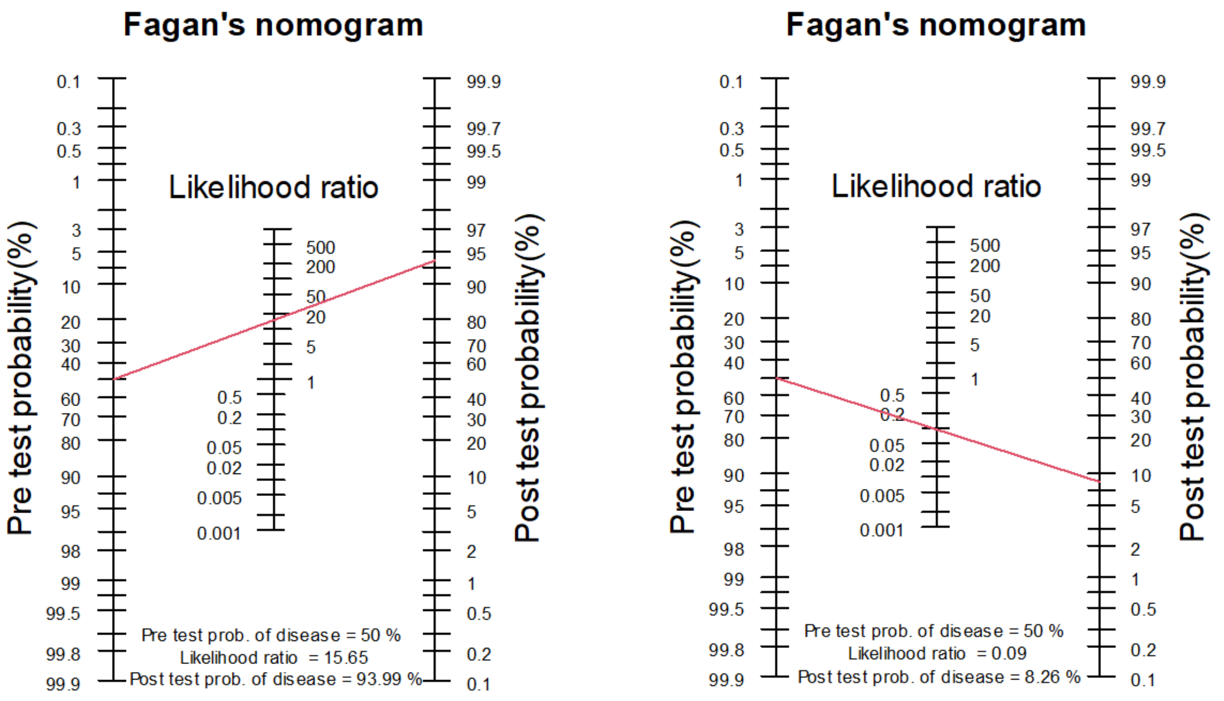

Figure S15 Fagan nomogram calculation of post-test probability with pre-test probability of 50%. Post-test probability is calculated for both a positive or negative result using the neural boosted model of dual scale StaphAIR array predictive variables plus albumin. This model had a LR+ of 15.65 and LR− of 0.09. The test could add clinical value for *S. aureus* diagnosis in individual patients.
